# Supplementary material for: Seasonality of the Meridional Overturning Circulation in the subpolar North Atlantic
Source: Commun Earth Environ. 2023 May 25;4(1):181. doi: 10.1038/s43247-023-00848-9 (PMC10211299; doi:10.1038/s43247-023-00848-9)
Supplement: Supplementary file 1 — Supplementary Information [file 43247_2023_848_MOESM1_ESM.pdf]

Supplementary Materials for

Seasonality of the Meridional Overturning Circulation in the Subpolar North Atlantic

Yao Fu, M. Susan Lozier, Tiago Carrilho Biló, Amy S. Bower, Stuart A. Cunningham, Frédéric Cyr, M. Femke de Jong, Brad deYoung, Lewis Drysdale, Neil Fraser, Nora Fried, Heather H. Furey, Guoqi Han, Patricia Handmann, N. Penny Holliday, James Holte, Mark E. Inall, William E. Johns, Sam Jones, Johannes Karstensen, Feili Li, Astrid Pacini, Robert S. Pickart, Darren Rayner, Fiammetta Straneo, Igor Yashayaev

Corresponding Author: Yao Fu (yaofu@gatech.edu) and M. Susan Lozier (susan.lozier@gatech.edu)

Table of content

Supplementary Figures 1 and 2

Supplementary Tables 1 to 3

Supplementary References 1 and 2

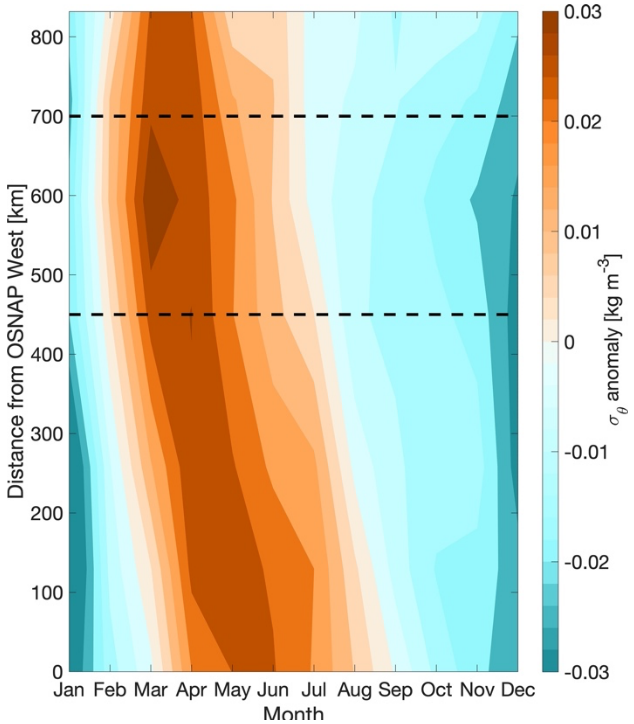

Supplementary Figure 1 Seasonal propagation of potential density anomaly along the Labrador Sea western boundary. Potential density between 200 and 400 m, calculated using Roemmich-Gilson gridded Argo data, is horizontally averaged within 2°×1° (longitude×latitude) bins centered on the 2500-m isobath. Potential density anomaly is calculated by subtracting the mean potential density of each bin. The 200-400 m range covers the depth range of the time-mean σ_{MOC} (27.69 kg m^{-3}) at OSNAP West, which has a mean depth of about 270 m along the western boundary. Y-axis represents the distance northward from OSNAP West. The two black dashed lines, one at 700 km and the other at 450 km, represent the approximate positions of 58°N and the AR7W hydrographic section (55°N) along the western boundary, respectively, which bracket the core of the climatological convection region in the Labrador Sea[1,2].

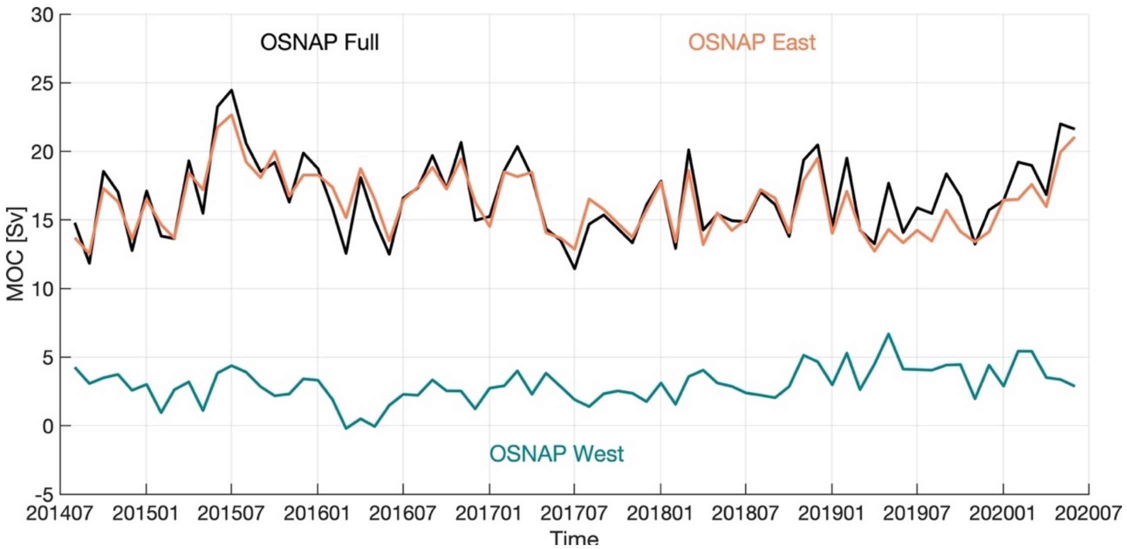

Supplementary Figure 2 Monthly MOC time series with the seasonal cycle removed. The climatological monthly anomaly derived from the 6-year OSNAP observations is removed from the original OSNAP MOC time series across the full OSNAP array (black), OSNAP East (orange), and OSNAP West (cyan).

Supplementary Table 1 Annual mean MOC across the OSNAP full array, OSNAP East and OSNAP West. The annual mean values are calculated from August to July of the next year. Note that the last available monthly value is in June 2020. Therefore, the values in the last column are derived based on 11 months observation from August 2019 to June 2020. The annual mean and standard error of the mean are derived based on the monthly values within the corresponding annual period. The corresponding standard deviation is shown in parenthesis.

Section	Aug 2014-Jul 2015	Aug 2015-Jul 2016	Aug 2016-Jul 2017	Aug 2017-Jul 2018	Aug 2018-Jul 2019	Aug 2019-Jun 2020
Full array	16.8±2.1 (5.4)	17.0±1.0 (2.6)	16.8±1.4 (3.5)	15.3±1.2 (3.0)	16.3±1.0 (2.5)	17.7±1.9 (4.7)
OSNAP East	16.5±2.0 (4.0)	17.3±1.1 (2.3)	16.6±1.4 (2.7)	15.3±1.2 (2.3)	15.4±0.8 (1.6)	16.3±2.0 (4.0)
OSNAP West	3.0±1.0 (1.1)	2.0±0.7 (0.8)	2.7±1.2 (1.3)	2.6±1.3 (1.4)	3.9±1.8 (2.0)	3.9±1.2 (1.3)

Supplementary Table 2 Climatological monthly MOC over the 6-year OSNAP observations. The climatological monthly mean and the standard error is provided for each month across the full OSNAP array, OSNAP East and OSNAP West. The value in parenthesis indicates the climatological monthly MOC with the Ekman transport component removed. Note that both the Ekman transport and the Ekman return flow are removed (see Methods).

Section	Jan	Feb	Mar	Apr	May	Jun	Jul	Aug	Sep	Oct	Nov	Dec
Full array	14.2±0.6<br>(17.8±1.1)	15.8±1.2<br>(19.2±1.0)	17.5±1.4<br>(19.8±1.1)	19.4±1.0<br>(19.6±1.4)	21.1±1.2<br>(20.8±1.2)	19.0±1.9<br>(19.0±1.9)	16.3±2.1<br>(16.5±1.9)	17.2±1.0<br>(17.6±1.1)	15.0±1.2<br>(16.3±1.0)	17.3±0.9<br>(18.4±0.7)	14.8±1.2<br>(16.2±1.3)	12.3±1.2<br>(14.9±0.9)
OSNAP East	15.9±0.7<br>(18.9±1.1)	16.7±0.8<br>(19.2±0.8)	16.8±0.9<br>(18.9±0.5)	18.9±1.1<br>(19.1±1.5)	19.2±0.9<br>(19.1±1.2)	17.8±1.6<br>(17.8±1.7)	14.7±1.7<br>(14.9±1.6)	16.5±1.0<br>(16.8±1.6)	15.2±0.9<br>(16.1±0.8)	15.7±1.0<br>(16.7±1.0)	14.3±1.0<br>(15.3±1.3)	13.0±0.9<br>(15.3±0.9)
OSNAP West	2.2±0.1<br>(2.8±0.2)	2.7±0.8<br>(3.6±0.8)	4.0±0.8<br>(4.4±0.7)	3.8±0.6<br>(3.8±0.5)	4.4±1.0<br>(4.3±0.8)	3.3±0.4<br>(3.4±0.4)	3.0±0.5<br>(3.1±0.4)	2.4±0.5<br>(2.5±0.5)	1.8±0.3<br>(2.2±0.3)	2.7±0.3<br>(2.9±0.3)	3.0±0.5<br>(3.4±0.4)	2.8±0.6<br>(3.3±0.5)

Supplementary Table 3 Climatological monthly Ekman transport over the 6-year OSNAP observations. The climatological monthly mean and standard error are provided for each month across the full OSNAP array, OSNAP East and OSNAP West.

Section	Jan	Feb	Mar	Apr	May	Jun	Jul	Aug	Sep	Oct	Nov	Dec
Full array	-4.0±0.9	-3.8±1.1	-2.7±1.1	-0.5±0.4	-0.1±0.8	-0.3±0.2	-0.6±0.3	-0.8±0.3	-1.7±0.4	-1.5±0.5	-1.7±0.9	-3.0±1.2
OSNAP East	-3.3±0.8	-2.9±0.9	-2.3±0.7	-0.4±0.4	-0.1±0.5	-0.2±0.1	-0.4±0.2	-0.6±0.2	-1.2±0.2	-1.2±0.4	-1.2±0.8	-2.5±1.0
OSNAP West	-0.7±0.2	-0.9±0.2	-0.4±0.4	-0.1±0.1	0.0±0.2	-0.1±0.1	-0.2±0.2	-0.1±0.1	-0.5±0.2	-0.3±0.1	-0.5±0.3	-0.5±0.2

Supplementary References

1. Yashayaev, I. & Loder, J. W. Further intensification of deep convection in the Labrador Sea in 2016. *Geophysical Research Letters* **44**, 1429–1438 (2017).
2. Li, F. & Lozier, M. S. On the linkage between Labrador Sea Water volume and overturning circulation in the Labrador Sea: A case study on proxies. *Journal of Climate* **31**, 5225–5241 (2018).
